# Evaluation of Nucleic Acid Stabilization Products for Ambient Temperature Shipping and Storage of Viral RNA and Antibody in a Dried Whole Blood Format

**DOI:** 10.4269/ajtmh.15-0110

**Published:** 2015-07-08

**Authors:** Allison L. Dauner, Theron C. Gilliland, Indrani Mitra, Subhamoy Pal, Amy C. Morrison, Robert D. Hontz, Shuenn-Jue L. Wu

**Affiliations:** Naval Medical Research Center, Silver Spring, Maryland; U.S. Naval Medical Research Unit No. 6 (NAMRU-6), Lima, Peru; Department of Entomology and Nematology, University of California, Davis, California

## Abstract

Loss of sample integrity during specimen transport can lead to false-negative diagnostic results. In an effort to improve upon the status quo, we used dengue as a model RNA virus to evaluate the stabilization of RNA and antibodies in three commercially available sample stabilization products: Whatman FTA Micro Cards (GE Healthcare Life Sciences, Pittsburgh, PA), DNAstāble Blood tubes (Biomātrica, San Diego, CA), and ViveST tubes (ViveBio, Alpharetta, GA). Both contrived and clinical dengue-positive specimens were stored on these products at ambient temperature or 37°C for up to 1 month. Antibody and viral RNA levels were measured by enzyme-linked immunosorbent assay (ELISA) and quantitative reverse transcription polymerase chain reaction (qRT-PCR) assays, respectively, and compared with frozen unloaded controls. We observed reduced RNA and antibody levels between stabilized contrived samples and frozen controls at our earliest time point, and this was particularly pronounced for the FTA cards. However, despite some time and temperature dependent loss, a 94.6–97.3% agreement was observed between stabilized clinical specimens and their frozen controls for all products. Additional considerations such as cost, sample volume, matrix, and ease of use should inform any decision to incorporate sample stabilization products into a diagnostic testing workflow. We conclude that DNAstāble Blood and ViveST tubes are useful alternatives to traditional filter paper for ambient temperature shipment of clinical specimens for downstream molecular and serological testing.

## Introduction

Accurate diagnosis of infectious diseases is needed to inform timely treatment and often requires temperature-controlled transportation of clinical specimens from the primary clinic to a diagnostic laboratory, conditions that may be unavailable in resource-limited environments. Failure to stabilize the specimen during transport could lead to false-negative diagnostic results, particularly for assays requiring viral RNA detection, in part due to the ubiquity of ribonucleases.^1^,^2^ Recent studies have demonstrated variability in current shipping conditions both in terms of expediency and temperature exposure of the sample.^3^,^4^ From these studies, one may conclude that sample stabilization prior to transport may be crucial to ensure accuracy of downstream diagnostic testing. To facilitate sample collection and storage in austere environments, such as those experienced during outbreaks of tropical diseases, a stabilization product ideally needs to be inexpensive as well as easy to use, requiring minimal ancillary equipment. In addition, the disease biomarkers in the specimen need to be preserved through the time and conditions to which they will be exposed during transport. Finally, the stabilization method needs to be compatible with all tests likely to be necessary for diagnosis, including molecular assays and serology.

In this study, we used dengue virus (DENV) as a model RNA virus for testing clinical specimen stabilization products for their ability to preserve nucleic acid and antibody in a dried blood format. Dengue is the most prevalent arthropod-borne viral disease, with four antigenically distinct serotypes: DENV1–4. Accurate diagnosis of dengue fever often requires both serological and molecular detection methods to distinguish it from other co-circulating fever-causing infectious agents, making DENV a good candidate for this evaluation. One of the most cost-effective and widely used methods of stabilizing whole blood for future testing is to create a dried blood spot on filter paper such as the Whatman FTA Micro Card (GE Healthcare Life Sciences, Pittsburgh, PA). FTA Micro Cards contain proprietary chemicals that protect nucleic acids from nucleases, as well as ultraviolet and oxidative damage. Many groups have examined the utility of dried blood spots on various types of filter paper for antigen, antibody, and nucleic acid–based diagnostics,^5^–^8^ especially for viruses of public concern such as the human immunodeficiency virus (HIV).^9^–^14^ This method is generally considered effective for stabilizing DNA for downstream molecular diagnostic applications.^15^ Despite their long-standing and widespread usage, diagnostic accuracy from dried blood spots can be influenced by many factors including original antibody or nucleic acid amount, hematocrit, size of the blood spot, and elution protocol used.^16^–^19^ Several products have recently entered the market, which may address these shortcomings. Using a proprietary technique based on anhydrobiosis involving a thermostable dissolvable glass, Biomātrica tubes (Biomātrica, San Diego, CA) “shrink-wrap” DNA in whole blood or purified RNA for long-term ambient temperature storage. Biomātrica DNAstāble has been shown to be effective for stabilizing DNA, but no previous studies have evaluated its ability to stabilize RNA in whole blood.^20^,^21^ Another manufacturer, ViveBio (Alpharetta, GA), has developed ViveST tubes that are claimed to stabilize RNA in dried plasma in a synthetic matrix, thus eliminating the need for upfront extraction. Studies using ViveST tubes have demonstrated hepatitis B, hepatitis C, and HIV nucleic acid stability ranging from 10 to 56 days following storage at ambient temperature.^22^–^24^

We evaluated these three commercially available specimen stabilization products for their ability to stabilize dengue viral RNA and anti-dengue immunoglobulin. We first tested these products over a period of 4 weeks at ambient and 37°C temperatures using contrived positive specimens created by spiking whole blood with DENV as well as human anti-dengue IgG. The products were then evaluated using clinical specimens collected and shipped from a dengue-endemic region of South America. RNA and antibody levels were tested using real-time reverse-transcription polymerase chain reaction (qRT-PCR) and enzyme-linked immunosorbent assay (ELISA), respectively, to evaluate the effect of time, storage temperature, and stabilization method on sample integrity. Together, this study represents a comprehensive laboratory and field evaluation of currently available sample stabilization matrices that may represent viable alternatives to dried blood spots.

## Materials and Methods

### Human use statement.

The procedures applied in this study were performed in accordance with the ethical standards of the Naval Medical Research Center (NMRC) Institutional Review Board (IRB) and with the Helsinki Declaration of 1975, as revised in 1983. Serum from dengue-positive patients was collected from febrile patients, who had provided permission for the future use of their blood samples, presenting to 12 health facilities within or near Iquitos, Peru, as part of an ongoing febrile surveillance study.^25^ Study protocols were approved by NMRC and the U.S. Naval Medical Research Unit No. 6 (NAMRU-6) IRB in compliance with all applicable federal regulations governing the protection of human subjects (NMRC.2005.0007 and NAMRU6.2014.0015).

### Sample preparation and stabilization.

#### Contrived DENV-positive samples.

DENV-positive samples were modeled by spiking commercially available CPDA-1-treated whole blood (Valley Biomedical, Winchester, VA) with tissue culture–derived DENV1 (West Pac-74) to a final titer of 10^4^ plaque-forming units (PFU)/mL and anti-dengue IgG-positive human plasma pooled from de-identified donors.

#### Clinical DENV-positive specimens.

Whole venous blood was collected into purple-top ethylenediaminetetraacetic acid (EDTA) tubes during ongoing surveillance studies from febrile individuals reporting to 12 health facilities in Iquitos. Samples were collected between 07:30 am and 1:00 pm and stored at 4°C until they were transported on ice to the NAMRU-6 field laboratory for processing by 2:00 pm. Thirty-seven specimens were confirmed to be dengue positive by the SD BIOLINE Dengue Duo (Dengue NS1 Ag and IgG/IgM) immunochromatographic rapid test (Alere Inc., Waltham, MA); dengue infection was later confirmed by RT-PCR and qRT-PCR as described elsewhere.^26^,^27^

#### Contrived sample application to stabilization products.

Contrived samples were applied to the products using the following method: 200 μL into DNAstāble Blood tubes (catalog no. 93027-027) followed by 15 minutes of shaking at 600–800 rpm, 1 mL onto ViveST tubes (catalog no. VSL2RL), and 200 μL onto Whatman FTA Micro Cards (catalog no. WB120210). All products were dried overnight in a biological safety cabinet, and then either eluted the following day (day 0) or placed at ambient temperature (~22°C) or 37°C until elution on days 1, 2, 7, 14, and 28. Aliquots of the original contrived whole blood were also frozen at −80°C to serve as a frozen unloaded positive control. Six to eight replicates for each product were generated for each time point and temperature combination.

#### Clinical sample application to stabilization products.

Clinical samples from Iquitos were applied to the products as described above, except that a matching 200 μL aliquot was frozen at −80°C as a frozen unloaded matched control, and only 500 μL was applied to the ViveST tubes due to sample volume restrictions. All products were dried overnight in a biological safety cabinet. The products were held at ambient temperature for up to 7 days before shipping. The stabilized specimens were then shipped at ambient temperatures from Iquitos to Lima, Peru, where they were held for an additional 5–8 days before elution and testing. Two shipments were required, and temperature and humidity data loggers (catalog no. 2C\TEMP-RH; Marathon Products, San Leandro, CA) were included in both shipments. The frozen unloaded matched controls were shipped in parallel on dry ice.

### Sample elution and extraction.

#### Elution.

The DNAstāble Blood tubes were reconstituted according to manufacturer’s recommendations. In brief, 300 μL of molecular grade water was added to each DNAstāble Blood tube at the appropriate time point, and the tube agitated at 1,000–1,200 rpm for 1 hour at ambient temperature. The ViveST tubes were also eluted according to manufacturer’s recommendation: the matrix was placed into a sealed standard, commercially available 3-mL syringe, after which 1 mL of water was added to the matrix and the syringe gently shaken to distribute the water. After 10 minutes, the plunger was used to expel all volume into a 1.5-mL microcentrifuge tube. FTA Micro Cards were eluted as described previously^14^: one-fourth of the blotted area was minced into small pieces, which were then placed into a 1.5-mL microcentrifuge tube. Nucleic acid was then eluted by adding 300 μL AVL buffer (catalog no. 19073; QIAgen, Valencia CA) to each tube and shaking for 1 hour at ambient temperature at 1,000–1,200 rpm, while antibodies were eluted by adding 300 μL of 0.1% PBS-Tween^®^ 20 (Corning CellGro, Manassas, VA; Fischer Scientific, Fair Lawn, NJ) to each tube and holding them overnight at 4°C. All eluates were then frozen at −80°C until the end of the study.

#### RNA extraction.

Using either a QIAgen EZ1 Advanced XL automated extractor (contrived samples) or QIAgen QIAamp viral RNA Mini kits (clinical specimens), 140 μL eluate was extracted according to the manufacturer’s protocols. Samples extracted using the QIAgen EZ1 Advanced XL were first diluted to 400 μL with 260 μL ATL buffer (catalog no. 19076; QIAgen) as required by the EZ1 Virus Mini Kit v.2.0 (catalog no. 955134). RNA was eluted in a final volume of 60 μL for downstream molecular characterization using qRT-PCR.

### Copy number and antibody concentration determination.

The presence of DENV RNA was determined using a qRT-PCR protocol as previously described.^28^ All specimens were run using the Applied Biosystems 7500 Fast Dx Real-Time PCR system (Life Technologies, Carlsbad, CA) utilizing the TaqMan EZ RT-PCR Core Reagent Kit (catalog no. N8080236; Life Technologies) with primers and a FAM-BHQ-labeled probe from Integrated DNA Technologies (Coralville, IA). Viral copy number was determined by utilizing a quantitated standard curve of dengue armored RNA (catalog no. 42044; Asuragen, Austin, TX). Anti-dengue IgM and IgG antibodies were analyzed using the Dengue Virus IgM Capture DxSelect and Dengue Virus IgG DxSelect ELISA kits, respectively (catalog no. EL1500M and EL1500G; Focus Diagnostics, Cypress, CA). All optical densities (ODs) were read at 450 nm within 30 minutes of adding stop solution. Index values were calculated by dividing the experimental OD by that of a provided cutoff calibrator as described in the manufacturer’s instructions.

### Statistical analysis.

Statistical significance of log_10_ copy number or ELISA index values was determined using the Mann–Whitney test (http://www.socscistatistics.com/tests/mannwhitney/Default2.aspx). *P* values < 0.05 were considered significant. Analysis of the concordance between qRT-PCR results of frozen versus stabilized clinical specimens was done using Pearson’s correlation coefficient as well as the Bland–Altman mean-difference method.^29^

## Results

### Recovery of nucleic acids from stabilization products.

Three nucleic acid stabilization products were evaluated extensively (Table 1). Contrived samples were added to the products according to the manufacturer’s instructions, dried overnight, and then eluted as described in the section Materials and Methods. The contrived samples were also maintained at −80°C without application of any stabilization product, as a frozen unloaded positive control for each experiment. We first tested the amount of dengue RNA for day 0, immediately following the overnight drying step. DENV RNA copies were then measured using qRT-PCR and compared against their corresponding frozen unloaded positive control. Any changes observed at this time point are likely based on application and recovery of sample from the stabilization matrix. We found that application and elution from both DNAstāble Blood tubes and FTA Micro Cards lead to a decrease in detectable DENV RNA, from 5.17 ± 0.51 to 4.63 ± 0.66 (Mann–Whitney test, *P* = 0.046) and 5.17 ± 0.60 to 3.36 ± 0.15 (*P* = 0.012) log_10_ copies/mL, respectively (Table 2). The decrease in RNA recovered from ViveST tubes was not statistically significant, with 5.24 ± 0.57 log_10_ copies/mL in frozen unloaded whole blood compared with 4.92 ± 0.51 log_10_ copies/mL at day 0 (*P* = 0.103).

### Stabilization of nucleic acids and antibodies over time.

We next wanted to examine the effect of time and temperature on RNA stability when using stabilization products. Using contrived dengue-positive samples, we found that DNAstāble Blood and ViveST tubes were able to stabilize RNA at ambient temperature for at least 28 days with no significant loss in copy number when compared with day 0 (Table 2). RNA stabilized on DNAstāble Blood tubes was determined to be 4.63 ± 0.66 (day 0) versus 4.33 ± 0.68 (day 28) log_10_ copies/mL, while RNA stabilized on ViveST tubes was 4.92 ± 0.51 (day 0) versus 4.57 ± 0.58 (day 28) log_10_ copies/mL. There was a significant difference in DENV copy number from day 0 to day 28 when stabilized at ambient temperature using FTA Micro Cards (3.36 ± 0.15 versus 2.80 ± 0.12 log_10_ copies/mL, *P* = 0.005). When temperature was elevated to 37°C, all products demonstrated significant decreases in RNA recovery following 28 days of exposure (*P* < 0.05). The copy number declined from 4.63 ± 0.66 to 3.8 ± 0.67 log_10_ copies/mL for DNAstāble Blood tubes, 4.92 ± 0.51 to 3.79 ± 0.85 log_10_ copies/mL for ViveST tubes, and 3.36 ± 0.15 to 2.60 ± 0.23 log_10_ copies/mL on FTA Micro Cards (Table 2).

**Figure 1.**
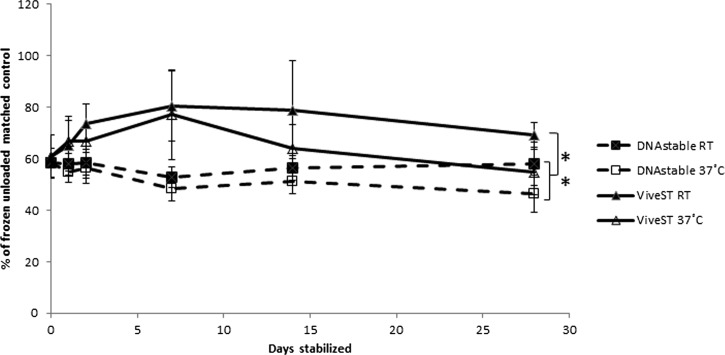
Anti-dengue IgG recovery over time. Anti-dengue IgG in contrived specimens was measured via enzyme-linked immunosorbent assay (ELISA) after stabilization for 0, 1, 2, 7, 14, and 28 days at ambient temperature or 37°C. Data are presented as a percentage of frozen unloaded positive control. Error bars represent standard deviation.

IgG titers for the same contrived dengue-positive samples that were dried in the blood tubes, were also stored for up to 28 days at ambient temperature or 37°C, and measured using a commercial ELISA at various time points. As with nucleic acid, a difference was observed between the index value of frozen unloaded positive controls and contrived samples stabilized on DNAstāble Blood or ViveST tubes at day 0 (1.32 ± 0.19 versus 0.74 ± 0.07 or 0.83 ± 0.12, respectively; *P* < 0.05). Antibody levels remain stable for at least 28 days on both products at ambient temperature when compared with day 0 (Figure 1

), whereas samples held at 37°C for 28 days were significantly different than those held at ambient temperature for the same period (*P* < 0.05).

### Shipment of clinical samples from field site to reference laboratory.

We next evaluated the products under real-world shipping conditions using clinical samples. EDTA-treated venous whole blood was collected from individuals with clinical suspicion of dengue and was then tested using the SD BIOLINE Dengue Duo rapid test. Whole blood from acute specimens testing positive for dengue nonstructural protein NS1 was then applied to the sample stabilization products and dried overnight as previously described. The specimens remained in the field laboratory for 0–7 days until they were sent in two shipments at ambient temperature to a centralized laboratory in Lima, where they remained for up to eight additional days prior to elution. During transport, the two shipments were exposed to temperatures ranging from 68.2 to 93.7°F (20.1–34.3°C), and relative humidity from 61.4% to 92.6% (Supplemental Figure 1). A total of 37 specimens were collected for this study, of which 36 were of the DENV2 serotype and 1 specimen remained uncharacterized for serotype due to low copy number.

**Figure 2.**
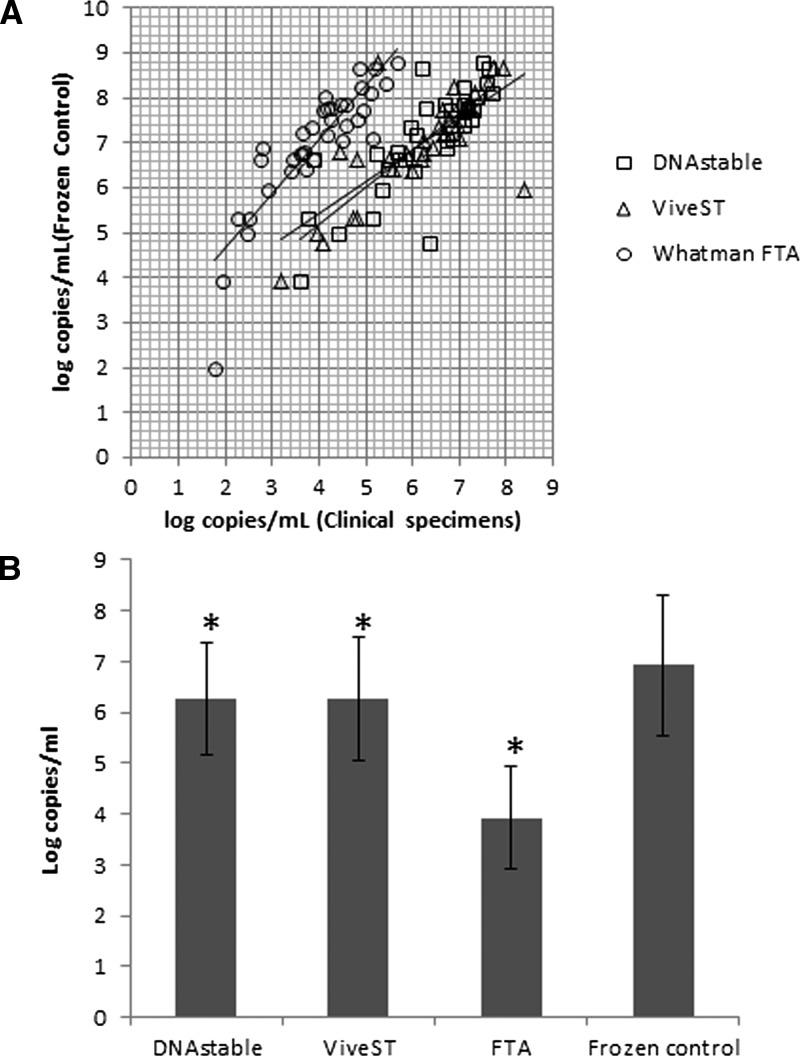
Comparison of dengue RNA recovery from clinical specimens shipped at ambient temperature vs. frozen unloaded matched controls. (**A**) Linear regression plots comparing dengue log_10_ copies/mL from stabilized clinical specimens vs. frozen unloaded matched controls. The correlation coefficients are as follows: DNAstable Blood *r* = 0.796; ViveST *r* = 0.752; FTA Micro Card *r* = 0.877. (**B**) Average log_10_ copies/mL ± standard deviation (SD) recovered (*N* = 36–37).

**Figure 3.**
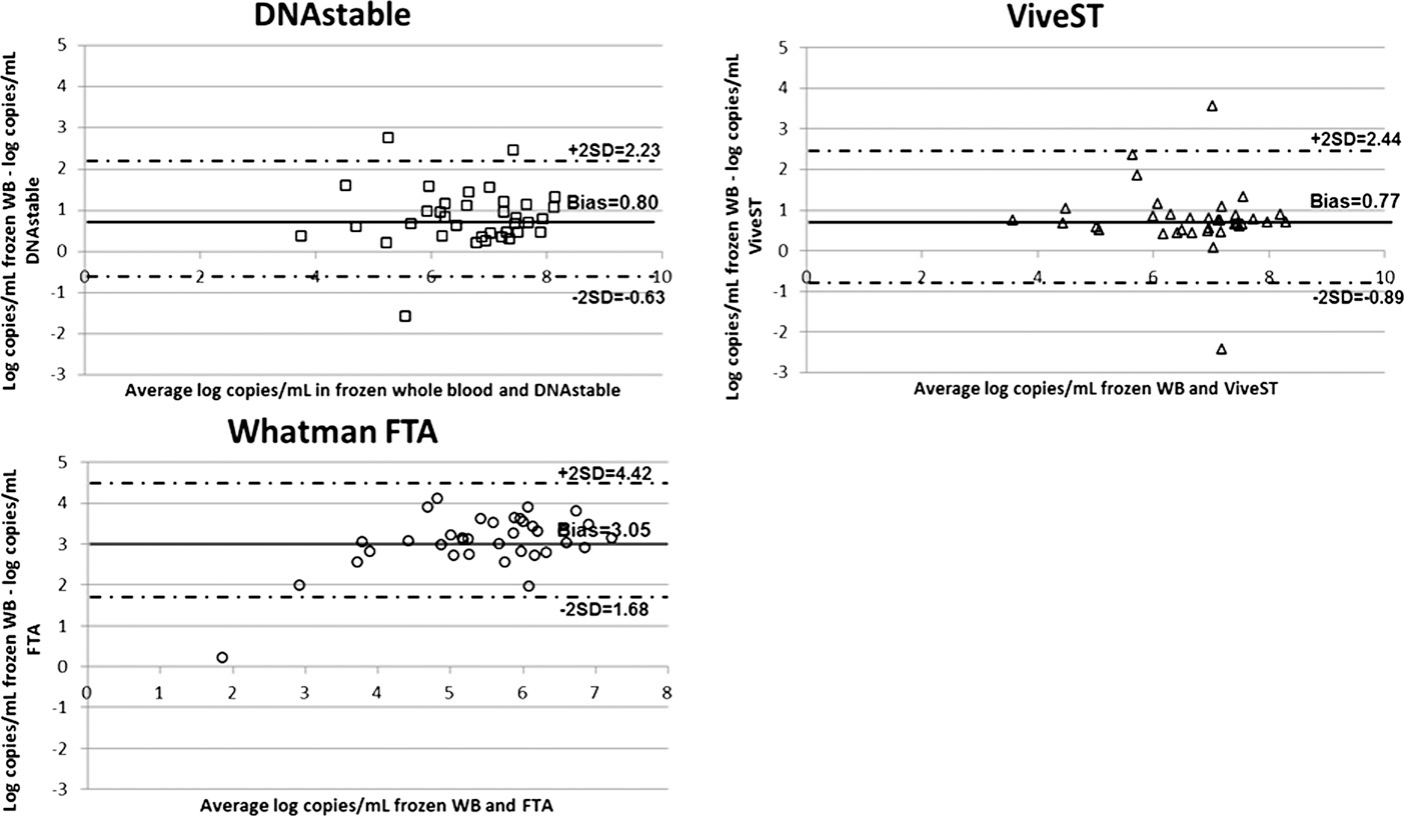
Bland–Altman mean-difference plots. Plots were generated for each product comparing viral RNA recovery between stabilized clinical specimens and frozen unloaded matched controls. The bias ± standard deviation (SD) are shown.

We first investigated the level of DENV RNA in specimens stored and transported at ambient temperature versus their frozen unloaded matched control by using qRT-PCR. There was a good correlation between stabilized specimens and frozen unloaded matched controls, with correlation coefficients of *r* = 0.796, 0.752, and 0.877 for specimens stabilized on DNAstāble Blood tubes, ViveST tubes, and FTA Micro Cards, respectively (Figure 2

). We found a significant difference in the average copy number between frozen unloaded matched controls and stabilized specimens, detecting 6.92 ± 1.4 log_10_ copies/mL from frozen unloaded matched controls compared with 6.26 ± 1.1, 6.26 ± 1.2, and 3.93 ± 1.0 log_10_ copies/mL for DNAstāble Blood tubes, ViveST tubes, and FTA Micro Cards, respectively (*P* < 0.01) (Figure 2B). Despite the observed decrease in log_10_ copies/mL, 36/37 (DNAstāble Blood tubes and FTA Micro Cards) or 35/37 (ViveST tubes) specimens were still qRT-PCR positive, leading to a percent agreement of 94.6–97.3%. Using Bland–Altman analysis (Figure 3

), we were able to calculate the mean difference between specimens stored at ambient temperature and their frozen unloaded matched controls. Specimens stabilized on DNAstāble Blood and ViveST tubes demonstrated a similar reduction in log_10_ copy number (0.80 and 0.77, respectively), while specimens eluted from FTA Micro Cards demonstrated significantly lower log_10_ copies/mL than the other two products (3.05, *P* < 0.01 using Mann–Whitney).

**Figure 4.**
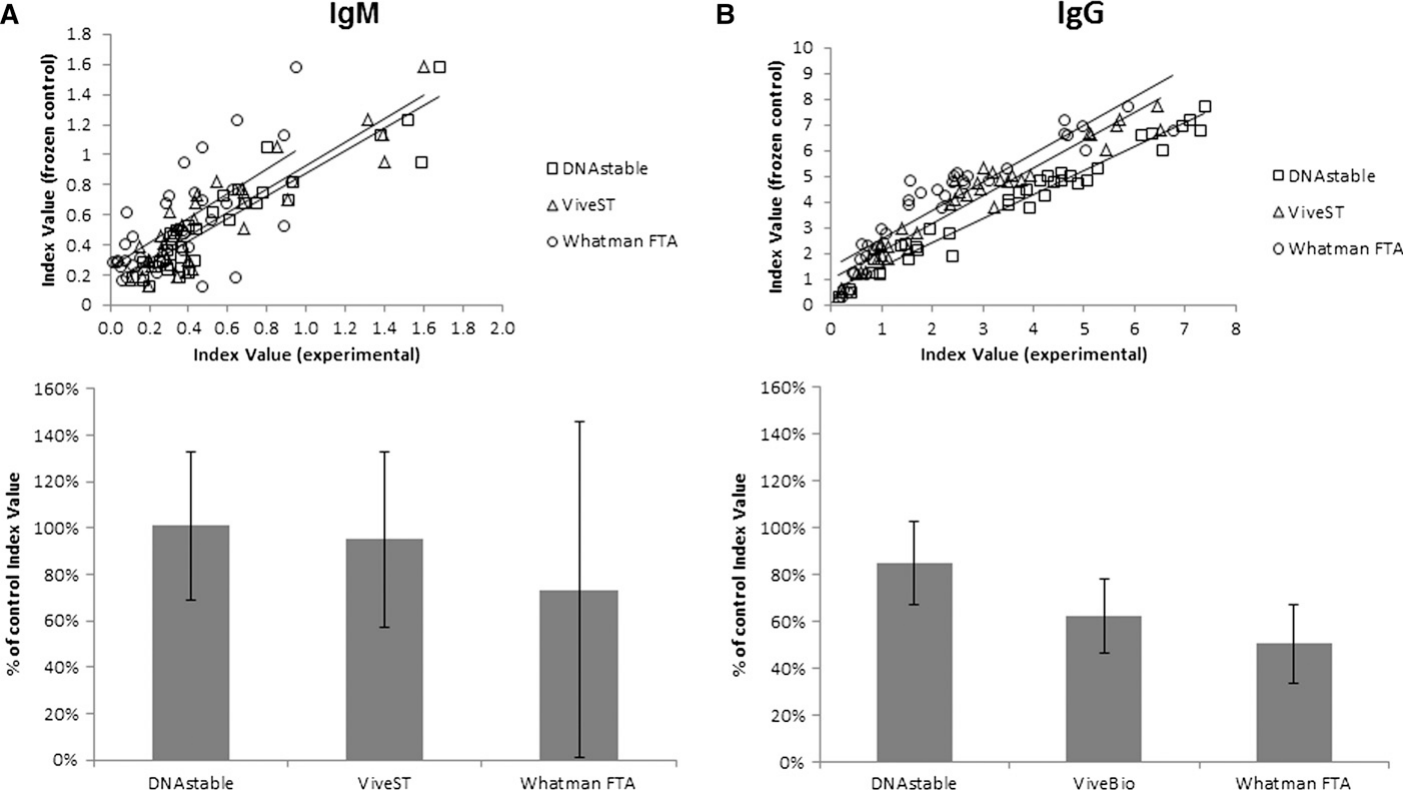
Comparison of IgM and IgG recovery from clinical specimens shipped at ambient temperature vs. frozen unloaded matched controls. Linear regression plots comparing index values from stabilized clinical specimens vs. frozen unloaded matched controls for anti-dengue (**A**) IgM or (**B**) IgG. Correlation coefficients for IgM are: DNAstable Blood *r* = 0.983; ViveST *r* = 0.979; FTA Micro Card *r* = 0.962. For IgG they are: DNAstable Blood *r* = 0.984; ViveST *r* = 0.966; FTA Micro Card *r* = 0.925. Average percent recovery ± standard deviation (SD) of anti-dengue (**A**) IgM or (**B**) IgG compared with frozen unloaded matched controls.

Next, we looked for the presence of anti-dengue IgM and IgG antibodies after stabilization and shipment, and compared the antibody levels to those in the frozen unloaded matched controls. As with nucleic acids, there was a strong correlation between clinical specimens shipped on each product and its frozen unloaded matched control (Figure 4A

). The Pearson’s correlation coefficients for IgM ELISA index values were 0.983, 0.979, and 0.962 for DNAstāble Blood tubes, ViveST tubes, and FTA Micro Cards, respectively. The levels of anti-dengue IgG were similarly correlated, wherein *r* = 0.984, 0.966, and 0.925, respectively. As a percent of frozen unloaded matched control, IgM recovered from FTA Micro Cards appeared lower than the other two products (73.3% versus 101% and 95.1% for DNAstāble Blood and ViveST tubes, respectively), with high variability (Figure 4B). Interestingly, the average percent recovery of IgG was lower than that seen for IgM, with DNAstāble Blood tubes demonstrating only 84.9% of frozen unloaded matched control followed by ViveBio (62.4%) and FTA Micro Cards (50.4%) (Figure 4B).

## Discussion

There are several scenarios that require preservation and transportation of biological samples, including research, diagnostic testing, or archival purposes. During infectious disease outbreaks, shipping samples for diagnostic testing becomes particularly urgent, as disease identification may be required to facilitate appropriate patient care and establish public health measures to curtail epidemic spread. In transporting samples from austere field environments to reference laboratories, dry ice or cold-chain shipment may not be available or may be prohibitively expensive. Further, using multiple sample stabilization methods may not be convenient, and one universal method is needed that is cheap, easy to use, and effective for stabilizing samples. To address these requirements, we began by evaluating the ability of three products, DNAstāble Blood tubes, ViveST tubes, and FTA Micro Cards, to stabilize RNA and antibodies over time, both at ambient and tropical temperatures using contrived samples. We then performed an evaluation using clinical specimens under real-world shipping conditions. Some of the products investigated were not intended to stabilize antibodies, or were specifically made to stabilize a specific nucleic acid (e.g., DNAstāble was not intended to stabilize RNA). However, we wanted to test these solutions for all targets, and also expose them to temperatures that they are likely to experience during shipment. Together, this represents a comprehensive evaluation using standardized panels of reagents and real-world shipping conditions, of sample stabilization solutions commercially available today that can still enable diagnostic testing for all relevant biological markers. DNA stabilization on these devices has been reported before, so we focused on measuring RNA and antibody levels. We were able to detect dengue virus RNA as well as anti-dengue antibodies from all products for at least 28 days, even when the products were held at 37°C. Some degradation of RNA was observed, especially when the samples were stored at higher temperatures. We conclude that all three products meet the target profile described earlier, and would be appropriate for short-term ambient temperature shipping from field clinics to diagnostic laboratories.

In addition to the products evaluated in this study, there are also commercially available RNA-stabilizing blood collection tubes such as RNAgard Blood tubes (Biomātrica), PAXgene Blood RNA (BD Biosciences, Oxford, UK), and Tempus Blood RNA tubes (Life Technologies). An advantage of this type of product is that the specimen can be collected directly into the stabilization tube, minimizing specimen handling and immediately stabilizing the nucleic acid. These products contain additives purported to stabilize RNA from degradation for 3–11 days at ambient temperature. Previous work in our laboratory confirmed their ability to stabilize RNA (data not shown), but since multiple stabilization techniques per sample is neither convenient nor cost-effective, we further examined these products for their ability to stabilize antibodies. These preliminary studies demonstrated an incompatibility of these tubes with downstream serological testing (i.e., universal false-positive anti-dengue IgG ELISA results).^30^ It is therefore important that the suitability of the specimen stabilization method with regard to the desired diagnostic testing be established prior to implementation.

Insufficient elution of clinical specimens from stabilization products can lead to a loss of diagnostic targets unrelated to time- or temperature-dependent degradation. Elution methods reported in the literature or recommended by manufacturer’s can also vary, especially for the Whatman FTA cards.^31^ Some methods may be better for eluting certain diagnostic targets than others. The loss can be partly ascribed to the volume of sample applied and eluted from the stabilization product. However, we found that this did not explain the extent of the loss in target that was observed. For example, 200 μL whole blood is dried into DNAstāble Blood tubes, followed by reconstitution in 300 μL, which can be represented as a dilution factor of 66% of the original concentration. Similarly, for ViveST tubes, where either 500 μL (clinical specimens) or 1,000 μL (contrived samples) were applied, followed by elution in 1,000 μL, the factor is 50% (clinical specimens) or 100% (contrived samples). Finally, only 50 μL (one-fourth of a 200 μL blot) of the FTA Micro Card is eluted into 300 μL, resulting in a factor of 17%. However, we observed an overall recovery rate of RNA on day 0 of 39% (DNAstāble Blood tubes), 49.8% (ViveST tubes), or 1.1% (FTA Micro Cards) of the frozen unloaded control. We hypothesize that adhesion may occur between the target (e.g., nucleic acid and/or antibodies) and the stabilization product reducing recovery, and that this may be particularly pronounced for the FTA Micro Cards. Reduced recovery may lead to inaccurate diagnostic results for specimens stabilized on FTA Micro Cards, particularly those containing lower viral titers. Our group has previously observed that low-titer DENV (10^2^ PFU/mL) stabilized on FTA Micro Cards was detectable by qRT-PCR for up to 1 month only about 50% of the time,^30^ and other groups have reported similar observations.^8^ Despite this loss, all products demonstrated a high-percent agreement using clinical samples in South America. This experiment was designed to capture a real-world shipping and testing scenario using patient samples, and a high agreement might suggest that the loss does not affect the detectability of most clinically relevant titers of dengue virus or antibody. In our study, shipments from Iquitos to Peru were received within 1 day, and it is likely that in other situations the samples may take longer to reach their destination. In future studies, it will be important to interrogate these products under more challenging conditions, including prolonged heat and humidity exposure.

Despite the observed reduction in RNA recovery, FTA Micro Cards remain one of the most cost-effective and versatile solutions for transporting whole blood samples. Unlike the other products, a finger stick can directly be applied to the FTA Micro Card to create a dried blood spot. The DNAstāble Blood and ViveST tubes, on the other hand, are likely to require trained phlebotomists to perform a venous blood draw. Advantages of DNAstāble Blood tubes include the simplicity of dried blood reconstitution and the self-contained nature of the tube, which is an important safety consideration during the transport of infectious agents. ViveST tubes are also self-contained, and benefit from being able to accommodate a larger volume of specimen (up to 1.5 mL). However, the elution procedure for ViveST tubes does require ancillary supplies (such as a syringe), potentially adding to the cost per specimen. All products recommend drying following sample application, which can take several hours and may delay specimen transportation and ultimately diagnostic results. One arena that would benefit greatly from incorporating simple and cost-effective sample stabilization is disease surveillance. In such studies, a large number of specimens are often collected weekly or monthly in austere environments with no access to subzero freezers, which can cost in excess of $10,000 each, exclusive of energy requirements that are an additional ∼$1,000/year.^15^ The suitability of each product for each specific application must be determined separately. Our data demonstrate that both ViveST and DNAstāble Blood tubes represent viable alternatives to dried blood spots, with unique advantages for storing human clinical specimens for downstream diagnostic testing such as qRT-PCR or ELISA.

## Supplementary Material

Supplemental Figure.

## Figures and Tables

**Table 1:** Stabilization product characteristics

Product	DNAstāble blood tubes	ViveST tubes	FTA micro cards
Company	Biomātrica	ViveBio	Whatman
Volume held	200 μL	1.5 mL	200 μL
Sample Recovery	• Add 300 μL sterile H_2_O	• Place matrix in syringe	• Mince ¼ spot
	• Shake at 1,200 rpm at RT, 1 hour	• Add 1 mL sterile H_2_O	• Add 300 μL AVL buffer
		• Incubate for 10 minutes	• Shake at 1,200 rpm at RT, 1 hour
		• Replace plunger and depress to recover	• Recover supernatant
Mechanism	Will stabilize DNA in whole blood in dry form using anhydrobiosis	Will stabilize plasma proteins and nucleic acids in dry form in synthetic matrix	Will stabilize proteins and nucleic acids in whole blood on chemically treated paper
Price	$4.50/tube	$5.00/tube	$2.93/card

RT = room temperature.

**Table 2:** Dengue virus RNA in contrived specimens after stabilization on three sample stabilization products for various time points and temperatures

Average log_10_ copies/mL ± SD (difference from frozen unloaded positive controls)						
	Day 0	Day 1	Day 2	Day 7	Day 14	Day 28
DNAstāble Blood RT	4.63 ± 0.66 (−0.54)	4.59 ± 0.71 (−0.58)	4.62 ± 0.59 (−0.55)	4.48 ± 0.51 (−0.69)	4.43 ± 0.43 (−0.73)	4.33 ± 0.68 (−0.84)
DNAstāble Blood 37°C	–	4.14 ± 0.61 (−1.02)	3.98 ± 0.44 (−1.19)	3.64 ± 0.51 (−1.53)	3.76 ± 0.45 (−1.41)	3.8 ± 0.67 (−1.37)
ViveST RT	4.92 ± 0.51 (−0.32)	4.65 ± 0.53 (−0.59)	4.44 ± 0.57 (−0.80)	4.24 ± 0.54 (−1.01)	4.24 ± 0.73 (−1.00)	4.57 ± 0.58 (−0.67)
ViveST 37°C	–	4.54 ± 0.53 (−0.71)	4.57 ± 0.55 (−0.67)	4.4 ± 0.59 (−0.85)	4.19 ± 0.67 (−1.05)	3.79 ± 0.85 (−1.45)
FTA Micro Card RT	3.36 ± 0.15 (−1.81)	3.24 ± 0.12 (−1.93)	3.26 ± 0.26 (−1.91)	3.1 ± 0.08 (−2.07)	2.93 ± 0.16 (−2.24)	2.8 ± 0.12 (−2.37)
FTA Micro Card 37°C	–	3.22 ± 0.18 (−1.95)	3.12 ± 0.16 (−2.05)	2.87 ± 0.50 (−2.30)	2.93 ± 0.17 (−2.24)	2.6 ± 0.23 (−2.57)

RT = room temperature.
