# Supplementary figures and images for: Evaluation of Nucleic Acid Stabilization Products for Ambient Temperature Shipping and Storage of Viral RNA and Antibody in a Dried Whole Blood Format
Source: Am J Trop Med Hyg. 2015 Jul 8;93(1):46–53. doi: 10.4269/ajtmh.15-0110 (PMC4497903; doi:10.4269/ajtmh.15-0110)

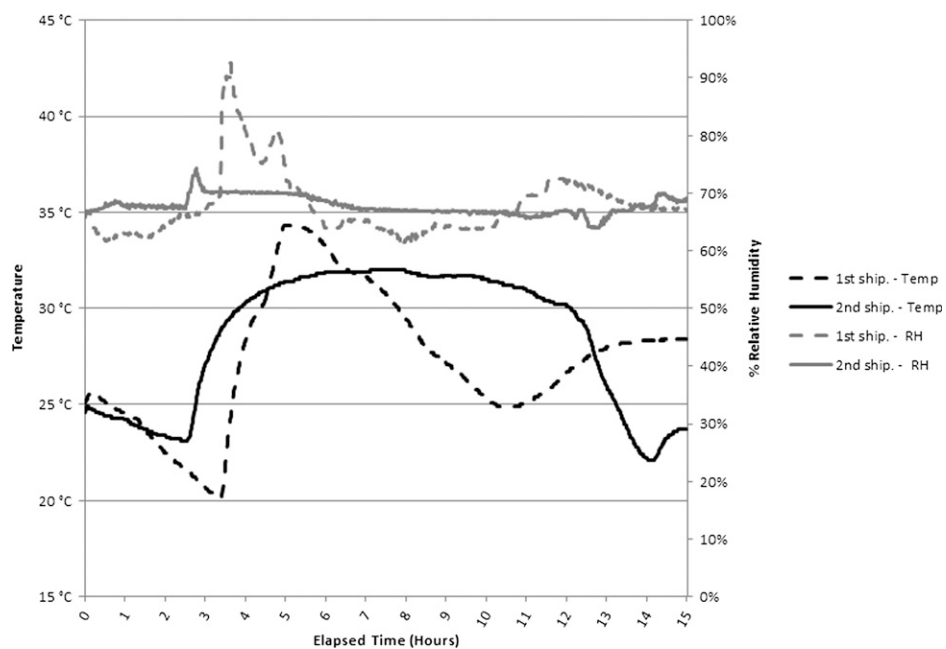

SUPPLEMENTAL FIGURE 1. Temperature and humidity ranges observed during shipment.

Supplement: Supplementary file 1 [file SD2.pdf]
